# Comparison of serum markers of inflammation in endometrioma and benign ovarian cysts

**DOI:** 10.61622/rbgo/2025rbgo58

**Published:** 2025-09-08

**Authors:** Arife Akay, Berna Dilbaz, Yaprak Engin-Üstün

**Affiliations:** 1 Yalova State Hospital Yalova Turkiye Yalova State Hospital, Yalova, Turkiye.; 2 Etlik Zubeyde Hanim Women's Health Education and Research Hospital Ankara Turkiye Etlik Zubeyde Hanim Women's Health Education and Research Hospital, Ankara, Turkiye.

**Keywords:** Endometrioma, Ovarian cysts, Endometriosis, Inflammation, Biomarkers, Postoperative period

## Abstract

**Objective:**

Endometriosis is known as a chronic inflammatory disease. This study investigates the differences in the inflammatory response between endometriomas and benign ovarian cysts during the preoperative and postoperative periods.

**Methods:**

A retrospective analysis was conducted on patients who underwent laparoscopic cystectomy for endometriomas or non-endometriotic benign cysts between 2010 and 2021. The study compared demographic and gynecological characteristics, lesion size, serum follicle-stimulating hormone (FSH), anti-Müllerian hormone (AMH), and preoperative and postoperative values of erythrocyte distribution width (RDW), neutrophil-lymphocyte ratio (NLR), platelet-lymphocyte ratio (PLR), and leukocytes between the two groups.

**Results:**

The study included 116 patients (48.13%) in the Study Group and 125 (51.87%) in the Control Group. The mean age of the Study and Control Groups was 27.76 years and 24.54 years, respectively (p<0.001). A non-significant discrepancy in preoperative AMH and FSH values was observed between the two groups (p>0.05). Preoperative RDW (14.50±1.56 vs. 14.04±1.40), PLR (160.82±44.52 vs. 136.83±48.72), and NLR (2.60±1.10 vs. 2.17±1.13) were significantly higher in the Study Group (p<0.05). Nevertheless, only in the Study Group NLR exhibited a notable increase in the postoperative period (p<0.05). Preoperative NLR was positively correlated with cyst size in endometriomas but was not correlated with rASRM score, AMH, or FSH levels.

**Conclusion:**

The inflammatory markers RDW, PLR, and NLR were significantly elevated in endometriomas compared to other benign cysts during preoperative and postoperative periods. The inflammatory response increased with cyst size but was not related to ovarian reserve as measured by serum AMH or the stage of endometriosis.

## Introduction

Endometriosis is an estrogen-dependent, chronic inflammatory disease with a multi-organ impact, which can cause pelvic pain and infertility.^(1)^ It is estimated that between 25% and 40% of women with infertility are affected by endometriosis.^(2)^ As with endometriomas, other ovarian cysts are relatively common occurrences, with 20% of women developing at least one pelvic mass throughout their lifetime.^(3)^

Endometriosis impacts metabolic processes in the liver and adipose tissue, triggers systemic inflammation, and alters gene expression in the brain, leading to increased pain sensitivity and the development of mood disorders.^(4)^ There is also evidence that women with endometriosis exhibit alterations in immune response, particularly a significant reduction in T-lymphocyte-mediated cytotoxicity against autologous endometrial cells.^(5)^ Emerging therapeutic options for endometriosis should address both the endocrine and immune system aspects of this complex gynecological syndrome.^(6)^ The systemic inflammatory response in endometriosis can result in changes in circulating leukocytes, manifesting as neutrophilia, thrombocytosis, and lymphocytopenia.^(7)^ Consequently, both the neutrophil-to-lymphocyte ratio (NLR) and the platelet-to-lymphocyte ratio (PLR) are often elevated in endometriosis.^(7)^

Despite the recognized nature of endometriosis as a chronic inflammatory disease, there remains a paucity of literature comparing the response in benign ovarian cysts both before and especially after surgery. This study investigates the differences in the inflammatory response between endometriomas and benign ovarian cysts in the pre- and post-operative periods. The present study also aims to contribute to the existing literature by demonstrating the relationship between ovarian reserve measured by serum anti-Müllerian hormone (AMH) and endometriosis stage assessment using the revised American Society for Reproductive Medicine (rASRM) score^(8)^ and serum inflammatory markers derived from complete blood count (CBC).

## Methods

A retrospective evaluation was conducted on patients who underwent laparoscopic cystectomy for endometriomas and non-endometriotic benign ovarian cysts between 2010 and 2021 at the Infertility Clinic of a tertiary center.

Patients aged 18-40 years who were operated on for ovarian cysts were included. The following exclusion criteria were applied: menopausal status, previous ovarian surgery, smoking, alcohol or substance addiction, presence of acute infections, pelvic inflammatory disease, chronic inflammatory diseases, comorbidities, preoperative and postoperative blood transfusion, intraoperative complications, ruptured cysts, and additional surgical procedures (e.g., myomectomy). Additionally, patients with presumed or histopathologically confirmed malignancy were excluded. A total of 542 cases that underwent laparoscopic cystectomy in our clinic between 2010 and 2021 were identified. A total of 301 cases were excluded from the study, including 72 cases with ovarian torsion and 69 cases with postmenopausal ovarian masses, 58 cases with ruptured non-endometrioma ovarian cysts, 35 cases with ruptured endometriomas, 27 cases under 18 years of age, 12 cases with malignant-borderline ovarian masses, and 28 cases that did not meet the inclusion criteria or had missing data. The Study Group involved cases with endometriomas, while the Control Group comprised women with non-endometriotic benign ovarian cysts, as determined by histopathology reports.

The data were extracted from the patient records or hospital records of all cases, including demographic and obstetric characteristics, the size of lesions, and the stage of endometriosis as determined by the rASRM score.^(8)^ Preoperative serum levels of follicle-stimulating hormone (FSH, mIU/mL), anti-Müllerian hormone (AMH, ng/mL), and cancer antigen 125 (CA-125, IU/mL) levels were quantified through the utilization of an enzyme-linked immunosorbent assay (ELISA, Roche®). CBC was performed using the Mindray BC-6000® device in our hospital's laboratory. CBCs were conducted preoperatively and on the first postoperative day for all patients. Serum inflammatory markers, including leukocytes (WBC), hemoglobin (HB), neutrophil-to-lymphocyte ratio (NLR), mean platelet volume (MPV), platelet-to-lymphocyte ratio (PLR), and erythrocyte distribution width (RDW) were obtained from the CBC.

A comparative analysis was conducted to examine the preoperative and postoperative serum levels of NLR, PLR, and RDW in the two groups. Moreover, delta (Δ) values, which indicate the alteration in each marker between the preoperative and postoperative stages, were contrasted between the groups. This study investigates the potential relationships between serum inflammatory markers and various variables, including lesion size, rASRM score, FSH, AMH, and CA-125.

The statistical analyses were conducted using the IBM SPSS Statistics software, version 21.0. The normality of numerical data was assessed using the Kolmogorov-Smirnov and Shapiro-Wilk tests. Numerical data are presented as mean ± standard deviation. Categorical variables were presented as numbers (percentages) and analyzed using the Chi-square (χ^2^) test. For variables with a normal distribution, a t-test was employed; for those with a non-normal distribution, a Mann-Whitney U test was utilized. The analysis of changes in markers within groups was conducted using a repeated measures ANOVA test, with delta (Δ) values calculated to represent changes. The Pearson correlation coefficient (r) was used to assess the relationship between normally distributed continuous variables. A receiver operating characteristic curve analysis was conducted to ascertain the 95% confidence interval (CI) values, cut-off values, sensitivity, and specificity values of markers with an area under the curve (AUC) exceeded 0.5. All statistical tests were conducted with a two-tailed hypothesis, and a p-value of less than 0.05 was considered statistically significant.

The study was conducted by the ethical standards set forth by the local ethics committee (approval number 2021/65, dated 09.06.2021).

## Results

A total of 241 cases that had definitive histopathology reports were included in this study. Of the total number of cases, 116 (48.13%) were assigned to the Study Group (endometrioma) and 125 (51.87%) were taken as the Control Group (non-endometriotic benign ovarian cysts). The Control Group exhibited a prevalence of 46.4% for mature cystic teratomas, 23.2% for serous cystadenomas, 16.8% for mucinous cystadenomas, and 13.6% for other benign ovarian cysts. The mean age of the Study Group was 27.76±5.38 years, which is higher than the mean age of the Control Group (24.54±6.08 years, p<0.001). A comparison of the BMIs of the two groups yielded results that were found to be similar (21.71 ± 4.02 vs 21.75 ± 4.00 kg/m^2^; p = 0.927). The nulligravida proportion was found to be 76.7% (n=89) in the Study Group, in contrast to the 80% (100) observed in the Control Group (p=0.214). Table 1 presents a comparative analysis of the sociodemographic and clinicopathological characteristics of the two groups.

**Table 1:** Comparison of demographic and clinical characteristics of the Study (endometrioma) and the Control (non-endometriotic benign ovarian cyst) groups

Variables		Study Group n= 116 48.13%	Control Group n=125 51.87%	p-value
Age (years), Mean ± SD		27.76±5.38	24.54±6.08	<0.001*
BMI (kg/m^2^), Mean ± SD		21.71±4.02	21.75±4.00	0.927*
Duration of İnfertilty (years), Mean ± SD		2.27±1.02	2.90±1.53	0.357**
Preoperative AMH (ng/ml), Mean ± SD		3.70±3.30	4.68±4.20	0.177**
Preoperative FSH (mIU/ml), Mean ± SD		6.91±2.61	6.08±2.53	0.079*
Preoperative CA-125 (IU/mL), Mean ± SD		13.88±292.43	19.52±19.71	<0.001**
Gravida	0	89 (76.7)	100 (80.0)	0.214***
	1	19 (16.4)	12 (9.69)	
	2-4	8 (0.9)	13 (10.4)	
Parity	0	94 (81.0)	107 (85.6)	0.038***
	1	21 (18.1)	12 (9.69	
	2-3	1 (0.9)	6 (4.8)	
Abortion	0	109 (94.0)	110 (88.0)	0.188***
	1	6 (5.2)	10 (8.0)	
	2-3	1 (0.9)	5 (4.0)	
Desire for fertility	None	53 (45.7)	78 (62.4)	0.027***
	Primary infertile	39 (33.6)	26 (20.8)	
	Secondary infertile	24 (20.7)	21 (16.8)	
Symptom on admission	Chronic pelvic pain	57 (49.1)	100 (80.0)	<0.001***
	Dysmenorrhea	44 (37.9)	21 (16.8)	
	Dysuria	7 (6.0)	2 (1.6)	
	Dyspareunia	7 (6.0)	2 (1.6)	
	Dyschezia	1 (0.9)	0 (0.0)	
Douglas obliteration	None	72 (62.1)	123 (98.4)	<0.001***
	Partial	11 (9.5)	2 (1.6)	
	Complete	33 (28.4)	0 (0.0)	
Presence of adhesion	None	35 (30.2)	120 (96.0)	<0.001***
	Thin-superficial	43 (37.1)	4 (3.2)	
	Dens-deep	38 (32.8)	1 (0.8)	
Lesion laterality	Unilateral	69 (59.5)	108 (86.4)	<0.001***
	Bilateral	47 (40.5)	17 (13.6)	
Total Size of the Cyst (cm), Mean ± SD		9.56±3.83	7.86±3.25	<0.001*
Right Size (cm), Mean ± SD		7.34±2.61	7.32±2.94	0.955*
Left Size (cm), Mean ± SD		6.13±2.83	6.43±2.85	0.543*
Duration of Operation (minutes), Mean ± SD		98.4±36	76.2±25.2	<0.001*

Bold is statistically significant at p <0.05;

* t-test;

** Mann Whitney U test;

*** Chi-square test; AMH - Anti-Müllerian hormone; BMI - Body mass index, CA125; Carcinogenic antigen 125; FSH - Follicle-stimulating hormone; SD - Standart Deviation

In cases with endometriomas, 49.1% were classified as stage 4 and 50.9% as stage 3 according to the rASRM staging system. The Douglas obliteration rate in the Study Group was significantly higher than in the Control Group (partial 9.5% vs 1.6%, complete 28.4% vs 0%, p<0.001). No statistically significant difference was identified between the groups with regard to preoperative AMH and FSH values (p > 0.05, Table 1). However, a significant elevation in preoperative CA-125 levels was observed in the Study Group versus the Control Group (132.88 ± 292.43 IU/mL vs. 19.52 ± 19.71 IU/mL, p < 0.001). A preoperative CA-125 cut-off value of 26.5 IU/mL demonstrated high sensitivity (83.2%) but low specificity (16.9%) for predicting the presence of endometrioma (95%CI: 0.843-0.931, AUC: 0.887). In the Study Group, the total cyst size (9.56 ± 3.83 vs. 7.86 ± 3.25 mm) was larger and the operation time (98.4 ± 36 vs. 76.2 ± 25.2 min) was longer than in the Control Group (p < 0.001). The comparison of preoperative and postoperative values of serum inflammatory markers, as well as the change (Δ) between the preoperative and postoperative periods, is presented in table 2. MPV and RDW values remained unchanged between the preoperative and postoperative periods in both groups. As demonstrated in table 2, while HB levels were observed to be lower (p < 0.001), the Study Group exhibited higher RDW, PLR, and NLR values (p = 0.019, <0.001, and 0.003, respectively). The Study Group exhibited statistically significant differences in NLR (2.72 vs 2.01, p=0.49). A weak positive correlation was identified between preoperative NLR and both WBC and cyst size in endometriomas (p<0.05, r=0.225 and 0.191, respectively). No correlation was observed between serum inflammatory markers and rASRM score, CA-125, AMH, or FSH values in the preoperative period (p>0.05).

**Table 2:** Comparison of preoperative and postoperative serum inflammatory markers and the changes (preoperative–postoperative, Δ) after the operation in the Study Group (endometrioma) and the Control Group (non-endometriotic benign ovarian cyst)

Variables	Study Group n=116 48.13%	Control Group n=125 51.87%	p-value
Preop. WBC (1/μl), Mean ±SD	7079.4±2189.65	7392.31±2321.92	0.284*
Preop. HB (gr/dl), Mean ±SD	12.29±1.19	12.84±1.01	<0.001*
Preop. PLT (1/μl), Mean ±SD	288.629±70.516	276.703±72,291	0.197*
Preop. MPV (fL), Mean ±SD	8.32±1.25	8.39±1.18	0.641*
Preop. RDW (%), Mean ±SD	14.50±1.56	14.04±1.40	0.019*
Preop. PLR (%), Mean ±SD	160.82±44.52	136.83±48.72	<0.001*
Preop. NLR (%), Mean ±SD	2.60±1.10	2.17±1.13	0.003*
Postop. WBC (1/μl), Mean ±SD	9914.48±3011.18	9415.52±2826.15	0.186*
Postop. HB (gr/dl), Mean ±SD	10.75±1.14	11.31±102	<0.001*
Postop. PLT (1/μl), Mean ±SD	251.232±64.253	244.040±59.673	0.368*
Postop. MPV (fL), Mean ±SD	8.33±1.22	8.36±1.17	0.868*
Postop. RDW (%), Mean ±SD	14.59±1.74	13.97±1.32	0.002*
Postop. PLR (%), Mean ±SD	179.18±69.89	146.48±56.36	<0.001*
Postop. NLR (%), Mean ±SD	5.31±2.91	4.18±2.21	<0.001*
Δ HB (gr/dl)	-1.54	-1.52	0.089**
Δ RDW%	0.09	-0.08	0.170**
Δ PLR %	18.36	9.65	0.289**
Δ NLR %	2.72	2.01	0.049**

Bold is significantly significant p<0.05,

* t-test;

** Repeated ANOVA test;

HB - Hemoglobin; MPV - Mean platelet volüme; NLR - Neutrophil lymphocyte ratio; PLT - Platelet count; PLR - Platelet lymphocyte ratio; Preop. - Preoperative period; Postop. - Postoperative period; RDW - Erythrocyte distribution width; SD - Standart Deviation; WBC - Leukocyte count; Δ - the difference between preoperative and postoperative values

**Figure 1:**
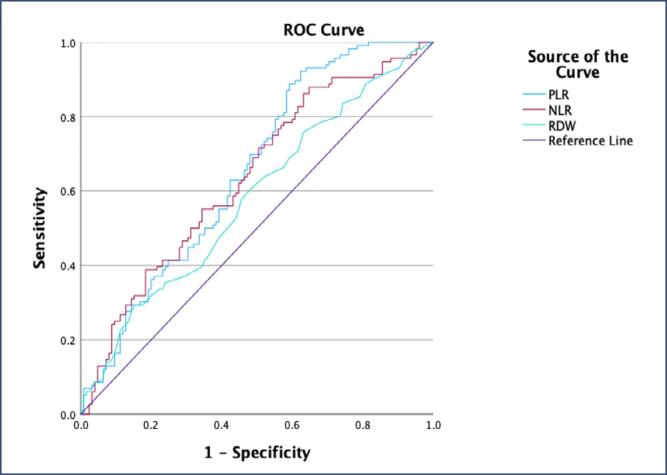
ROC curve of neutrophil-to-lymphocyte ratio (NLR), platelet-to-lymphocyte ratio (PLR), and erythrocyte distribution width (RDW) in the preoperative period

**Figure 2:**
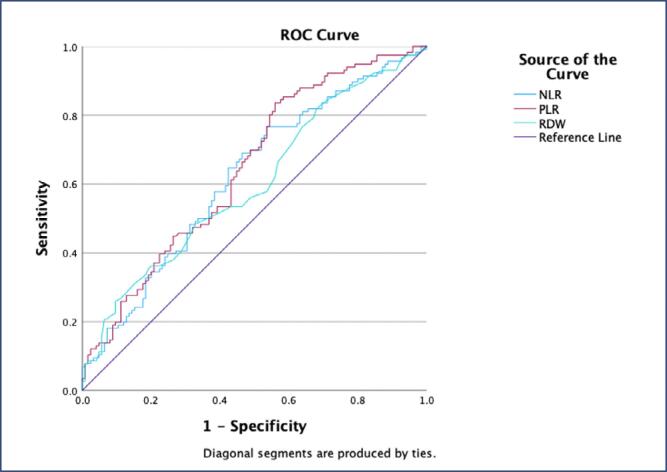
ROC curve of neutrophil-to-lymphocyte ratio (NLR), platelet-to-lymphocyte ratio (PLR), and erythrocyte distribution width (RDW) in the postoperative period

The preoperative NLR was 2.13, with 56.9% sensitivity and 56.8% specificity (AUC=0.638, p<0.001, 95%CI 0.568, 0.707) being identified as the optimal cut-off value. The postoperative NLR was 4.13, with 58.6% sensitivity and 58.4% specificity (AUC= 0.621, p<0.001, 95%CI 0.551-0.692) being identified as the optimal cut-off value. Furthermore, the ROC analysis results of PLR and RDW in the pre-and postoperative periods are presented in table 3, together with the cut-off values, sensitivities, and specificities. The ROC curves of NLR, PLR, and RDW are displayed both in figure 1 for the preoperative period and figure 2 for the postoperative period.

**Table 3:** The results of the ROC curve analysis of the neutrophil-to-lymphocyte ratio (NLR), platelet-to-lymphocyte ratio (PLR), and erythrocyte distribution width (RDW) in the preoperative and postoperative periods

Variables	AUC	p-value	Cut-off values	Sensitivity %	Specificity %	95% Confidence Interval	
						Lower bound	Upper bound
Preop. RDW	0.580	0.031	13.95	0.578	0.544	0.508	0.652
Preop. PLR	0.654	<0.001	141.44	0.586	0.584	0.585	0.722
Preop. NLR	0.638	<0.001	2.13	0.569	0.568	0.568	0.707
Postop.RDW	0.599	0.008	13.95	0.534	0.536	0.528	0.671
Postop. PLR	0.646	<0.001	151.46	0.569	0.568	0.577	0.715
Postop. NLR	0.621	0.001	4.13	0.586	0.584	0.551	0.692

p<0.05 is significantly significant. Analysis of receiver operating characteristic (ROC) curve. AUC - Area under the curve; NLR - Neutrophil lymphocyte ratio; PLR - Platelet lymphocyte ratio; Preop. - Preoperative period; Postop. - Postoperative period; RDW - Erythrocyte distribution width

The optimal RDW cut-off is 13.95, with a sensitivity of 57.8% and a specificity of 54.4%

The optimal PLR cut-off is 141.44, with a sensitivity of 58.6% and specificity of 58.4%

The optimal NLR cut-off is 2.13, with a sensitivity of 56.9 % and a specificity of 56.8 %

The optimal RDW cut-off is 13.95, with a sensitivity of 53.4% and a specificity of 53.6%

The optimal PLR cut-off is151.46, with a sensitivity of 56.9% and specificity of 56.8%

The optimal NLR cut-off is 4.13, with a sensitivity of 58.6 % and a specificity of 58.4 %

## Discussion

It has been demonstrated that women with endometriosis exhibit alterations in both the peripheral immune system and the immune status of the endometrium.^(9)^ The present study aimed to compare the inflammatory status of endometriomas with non-endometriotic benign ovarian cysts by utilizing serum inflammatory markers. The results indicated that NLR, PLR, and RDW were significantly higher in endometriomas in both preoperative and postoperative periods. However, the change in NLR between the preoperative and postoperative periods showed a significantly greater increase in endometriomas. Moreover, a significant association was observed between preoperative NLR and cyst size in endometriomas.

The functional receptors of macrophages are regulated by estrogen, indicating a relationship between estrogens and the immune response in endometriosis.^(10)^ Neutrophils and macrophages in the peritoneum secrete pro-inflammatory cytokines, including VEGF, IL8, and IL12, which can support the growth, invasion, and angiogenesis of endometriotic cells.^(11,12)^ The capacity of lymphocytes to destroy endometrial cells in the peritoneal cavity is limited, and the release of inflammatory substances induces the adhesion and migration of leukocytes through interactions between endothelial cells and platelets.^(5,11)^

In 2021, the study conducted by Moini et al.^(13)^ yielded comparable findings, with 54.8% of the study group exhibiting Stage 3 and 48.3% exhibiting Stage 4. The study group exhibited lower Hb levels and higher WBC, MPV, PLR, and NLR in their study.^(13)^ Similarly, our study observed a notable decline in Hb levels and a significant increase in NLR and PLR in endometriomas during the preoperative period. Ding et al.^(14)^ reported that serum CA-125 (sensitivity 80.6% and specificity 85.4%, cut-off level 27.03 IU/mL) and NLR (cut-off level 0.56%, sensitivity 32.9% and specificity 80.2%) were significantly elevated as biomarkers in patients with endometriosis. The study also demonstrated that NLR could be a potential neoadjuvant biomarker alongside serum CA-125 in the diagnosis of endometriosis. Similarly, our study determined sensitivity values for CA-125 and NLR at 83.2% and 56.9%, respectively, and specificity values of 16.9% and 56.8%, respectively.

Yang et al.^(15)^ reached the conclusion that PLR was significantly enhanced in patients suffering from moderate to severe endometriosis, achieving a sensitivity of 65%, a specificity of 51.8%, and a cut-off value of 117.16 IU/m. The present study revealed a significantly higher mean PLR in endometriomas (160.82±44.52) compared to the Control Group (136.83±48.72) (p<0.001). In a recent study, Kurt et al.^(16)^ observed a notable elevation in RDW levels amongst the moderate-to-severe Endometriosis Subgroup when compared to the mild-moderate Endometriosis Subgroup and the Control Group. Additionally, our study revealed that preoperative RDW was markedly elevated in the endometrioma cohort (p=0.019).

Although some studies^(12,17)^ have indicated a favorable relationship between the stage of endometriosis and inflammatory markers, particularly for NLR, this outcome has not been consistently corroborated by other studies.^(6,18,19)^ The study conducted by Kim et al.^(17)^ revealed no correlation between lesion size and NLR. The present study yielded no evidence of a link between the rASRM score and inflammatory biomarkers. Nevertheless, a positive correlation was identified between the total cyst size and the preoperative WBC and NLR values. It is established that NLR is elevated in certain malignancies, particularly epithelial ovarian cancer, where it reflects a robust systemic inflammatory response.^(20)^ An elevated postoperative NLR and PLR have been associated with advanced-stage ovarian malignancy.^(21)^ In the present study, the inter-period change was analyzed, and it was found that only the ΔNLR value was significantly higher in endometriomas compared to the control group.

The results of a systematic review of 572 patients across 14 studies indicated that systemic alterations in inflammatory markers have been linked to an elevated risk of cardiovascular and thrombotic complications in women with advanced endometriosis.^(22)^ This highlights the potential cardiovascular risk associated with these inflammatory changes.

One of the significant advantages of utilizing inflammatory response and alterations in blood parameters in this study is that these markers can be readily obtained from routine CBC. This study has several strengths, including the rigorous application of exclusion criteria, which effectively eliminated other factors that could influence the inflammatory process. This allowed for a more accurate observation of the inflammatory process in endometriosis. Additionally, the study explored the relationship between factors such as the stage of endometrioma and cyst size, which may be associated with inflammation, and these markers. However, a limitation of the study is the inability to evaluate early-stage endometriosis, as the majority of cases were at stages 3 and 4. This limitation is inherent to the retrospective design of the study. Moreover, given that this study was conducted in a single center, its generalisability is limited. Further randomized controlled studies are necessary to mitigate potential biases resulting from its retrospective design.

## Conclusion

Serum inflammatory markers, including NLR, PLR, and RDW, demonstrate a notable elevation in both the preoperative and postoperative periods in endometriomas compared to other benign ovarian cysts. However, the change in NLR between the preoperative and postoperative periods was significantly higher in endometriomas. Moreover, the findings of the present study demonstrated that NLR exhibited a significant positive correlation with cyst size in patients diagnosed with endometrioma. However, no correlation was observed between the NLR and AMH or FSH levels. In conclusion inflammatory response that demonstrates itself with a rise in blood-derived inflammatory biomarkers is amplified in larger endometriomas irrespective of the ovarian reserve. Future research is required to evaluate the significance of inflammatory biomarkers in the diagnosis of peritoneal endometriosis without the presence of an endometrioma particularly in the context of early-stage endometriosis. Thus, the precise role of serum inflammatory markers in the pathogenesis and prognosis of endometriosis requires further research.
